# Epimedium Flavonoids Counteract the Side Effects of Glucocorticoids on Hypothalamic-Pituitary-Adrenal Axis

**DOI:** 10.1155/2013/938425

**Published:** 2013-09-23

**Authors:** Jianhua Huang, Jijun Li, Songbai Zheng, Junzhen Wu, Wei Zhang, Tao Sun, Sheilesh Kumar Dewan, Bill Kalionis, Ziyin Shen, Xiantao Tai, Shijin Xia

**Affiliations:** ^1^Key Laboratory of Cellular and Molecular Biology, Huashan Hospital, Fudan University, Shanghai 200040, China; ^2^Department of Integrative Medicine, Shanghai Children's Medical Center, Shanghai Jiaotong University School of Medicine, Shanghai 200127, China; ^3^Department of Geriatrics, Huadong Hospital, Fudan University, Shanghai 200040, China; ^4^Department of Perinatal Medicine, The Royal Women's Hospital, University of Melbourne, Parkville, VIC 3052, Australia; ^5^School of Acupuncture, Massage and Rehabilitation, Yunnan University of Traditional Chinese Medicine, Kunming 650500, China; ^6^Shanghai Institute of Geriatrics, Huadong Hospital, Fudan University, Shanghai 200040, China

## Abstract

Our previous studies demonstrated that the epimedium herb, when simultaneously used with GCs, counteracted suppressive effects of GCs on the HPA axis without adverse influence on the therapeutic action of GCs. Here, total flavones were extracted from the epimedium flavonoids (EFs) and then used to investigate whether EFs provide protective effects on the HPA axis. We found that GCs induced a significant decrease in body weight gain, adrenal gland weight gain, and plasma adrenocorticotropin (ACTH) and corticosterone levels. After treatment with EFs, body weight gain, adrenal gland weight gain, and plasma corticosterone level were significantly restored, whilst plasma ACTH level was partially elevated. EFs were also shown to promote cell proliferation in the outer layer of adrenal cortex and to enhance the migration of newly divided cells toward the inner layer. To elucidate the underlying mechanisms, the mRNA expression of insulin-like growth factor II (IGF-II) was measured, and EFs significantly upregulated IGF-II expression. Our results indicated that EFs counteract the suppression of the HPA axis induced by GCs. This may involve both the ACTH and IGF-II pathways and thereby promote regeneration of the adrenal cortex suggesting a potential clinical application of EFs against the suppressive effects of GCs on the HPA axis.

## 1. Introduction

Glucocorticoids (GCs) are widely used and have been successful for more than 50 years in the treatment of some inflammatory or autoimmune diseases including asthma, nephrotic syndrome, lupus, and nephritis [1, 2]. Unfortunately, the beneficial effects are often accompanied by multiple undesirable adverse effects. These include diabetes mellitus, peptic ulcer, Cushing's syndrome, and osteoporosis. The use of GCs also results in suppression of hypothalamic-pituitary-adrenal (HPA) axis, which may persist after treatment withdrawal [3–5]. Full restoration of HPA function may require several months or, in some cases, more than one year [6].

The adverse effects of HPA suppression are particularly severe and correlate highly with the failure of GC withdrawal and reactivation of diseases. HPA suppression is the direct cause of the acute adrenal insufficiency syndrome, which can be precipitated by surgery or major stress, and is associated with the life-threatening condition of hemodynamic collapse [7]. Thus, there is a need for the development of compounds with a protective effect on the HPA axis, which can be used in combination with GCs.

There are several reports suggesting a potential therapeutic benefit of some agents for preventing the HPA axis suppression induced by the long-term use of GCs. Polypeptide pneumadyne (PNM) can elevate the content of corticosterone in adrenal tissue and increase cell volume and cell number in the adrenal, possibly by stimulating the release of arginine vasopressin (AVP) and adrenocorticotropic hormone (ACTH); however, PNM did not increase the serum level of corticosterone [7]. Buspirone, an antidepression drug and a 5-HT receptor agonist, was shown to enhance the synthesis and release of ACTH, suggesting an antagonistic role against the suppression of the HPA axis by dexamethasone, which was used in the treatment of depression [8]. Huang reported that ovine corticotrophin releasing hormone, vasopressin, and exogenous ACTH can partially restore the suppressed HPA function in animals [9]. However, none of these drugs have been used in clinical settings specifically to treat the suppression of the HPA axis.

An agent that not only resists the suppression of HPA axis induced by GCs, but also has no adverse influence on the therapeutic action of GCs is required. Our previous studies revealed that a herbal compound prescribed in traditional Chinese medicine and already used in clinical treatment may be a suitable agent [10]. Oral GCs were successfully used to control asthma, but due to their suppression of the HPA axis, GCs dependence was frequently developed. The successful withdrawal rate of GCs was very low, at about 12% [11, 12]. Subsequently, inhalation of large doses of GCs was used to attenuate the adverse effects on the HPA axis, and the successful withdrawal rate increased up to 27~44% [13, 14]. In order to further promote the success rate, GCs were used in combination with a herbal compound prescription, *Kidney*-tonifying decoction. Consequently, we found that the successful withdrawal rate was significantly increased to 70% [10].

The encouraging results from clinical studies prompted us to isolate the possible effective components through which the *Kidney*-tonifying decoction exerts protective efficacy on HPA axis. Among the numerous ingredients, we identified epimedium flavones (EFs) extracted from epimedium herb contained in the *Kidney*-tonifying decoction as a major component. We postulated that EFs were the effective components within the *Kidney*-tonifying decoction. In the present study, we examined the protective role of EFs on the HPA axis in animals treated with large doses of GCs.

## 2. Experimental Section

### 2.1. Study Design

Four-month-old male Sprague-Dawley rats weighting 200 ± 15 g were randomly assigned into 3 groups, that is a normal control group, a corticosterone only-treated group, and a combined corticosterone and EF -treated group. Each group consisted of 16 rats. All rats were housed in a temperature-controlled room (24°C) with lights on from 0600 h~1800 h daily and were allowed free access to water and rat pellet chow. The corticosterone only-treated group was subcutaneously injected with 0.1 mL corticosterone dissolved in olive pomace oil at a dose of 10 mg/kg body weight per day for 14 days. This group received simultaneous oral saline (0.2 mL) for 14 days. The combined corticosterone and EF-treated group was injected with the same dose of corticosterone as the corticosterone-treated group, but received EF orally at a daily dose of 60 mg/kg body weight. In the control group, rats were subcutaneously injected with 0.1 mL olive pomace oil and orally administrated with the same volume of saline as above. Fourteen days later, the rats were killed by decapitation. The animal protocols were in accordance with Animal Care and Use Committee of Fudan University.

### 2.2. Preparation of EFs

Epimedium is a major component in Kidney-tonifying decoction, which has been used to increase the success rate of GCs withdrawal. The Epimedium was purchased from a traditional chinese medicine distributor (Shanghai U-sea Biotech Co., Ltd., Shanghai, China). The origin of epimedium was in Liaoning province, China. The epimedium was verified by De-yun Kong, a professor of Shanghai Institute of Pharmaceutical Industry, China. A voucher specimen was deposited in the Herbarium of Shanghai Institute of Pharmaceutical Industry, China. Only the leaves and stems of Epimedium were used in our study. The total flavonoids of epimedium were extracted and used as previously [15]. Epimedium was ground to a powder (about 30 meshes) by a disintegrator, and the powder (1000 g) was extracted twice with 10 L 75% ethanol for 2 h under reflux. The extracts were combined together and filtrated with cotton, and the filtrate was concentrated under vacuum to give an aqueous fluid. The aqueous fluid was subjected to a glass column (5 cm × 80 cm) of macroporous resin (D101; 400 g) and washed with water and 70% ethanol, respectively. The eluent of 70% ethanol was concentrated in vacuum using a rotary evaporator to dry the EFs powder (11.5 g; purity >70%). The EFs were solvated with water to a 3% concentration before orally administered to the rats [15]. The content of ingredients of EFs was determined by high-performance liquid chromatography (HPLC).

### 2.3. Plasma ACTH Assay

After rats were sacrificed, blood samples of 2 mL per rat were immediately collected into heparin-pretreated tubes and tubes, were incubated for 30 min at room temperature followed by centrifugation at 1000 g for 15 min. The supernatant was obtained and aliquots of 0.15 mL were prepared and stored at −80°C prior to assay. The assay was performed using the DSL-10-5100 ACTIVE ELISA kit (Diagnostic Systems Laboratories, TX, USA). The detailed protocol was carried out according to the manufacturer's instructions. In brief, 100 *μ*L of various concentrations of ACTH standards, positive control, and samples were used, respectively, followed by addition of antibody-biotin conjugate, streptavidin-enzyme conjugate, chromogen solution, and stopping solution, in turn. Finally, all samples were assayed at wavelength of 405 nm, and a wavelength of 530 nm was used as a reference. 

In order to determine the interassay coefficient of variation (CV), three samples were assayed in the same experiment 16 times, then the CV was calculated. In order to determine intrassay CV, three samples were assayed in 8 separated experiments, and the CV value was calculated. 

### 2.4. Plasma Corticosterone Assay

The sample preparation was the same as the ACTH assay. For determining intra- or interassay CV, the same method as that in ACTH assay was used. The EIA kit for corticosterone assay was purchased from Cayman Chemical Company (MI, USA). In brief, 50 *μ*L of corticosterone standards and samples were used. The detection was consistent with the protocols provided by the manufacturer. Finally, all samples were assayed at wavelength of 412 nm.

### 2.5. Immunohistochemical Detection of 5-Bromo-2′-deoxyuridine (BrdU) Incorporated Nuclei

Twelve hours before rats were sacrificed, the rats were injected with 50 mg BrdU/kg body weight, 3 times at a time interval of 3 hours. The adrenal glands were excised, fixed in formalin overnight, and embedded in paraffin. Slides containing 4 *μ*m sections were prepared and subjected to 4 N HCL to denature the DNA. The sections were incubated with a mouse anti-BrdU antibody (Sigma) and then incubated with a biotinylated secondary antibody and horseradish peroxidase-conjugated streptavidin (Boster Co., Wuhan, China). Colour detection was by addition of 0.05% 3,3′-diaminobenzidine (Sigma) and 0.01% hydrogen peroxide at room temperature. Sections were then lightly counterstained with 0.1% hematoxylin. At least three fields in the outer layer of adrenal gland were chosen for assessment. The ratio of BrdU-positive cells versus total cell numbers defined by hematoxylin-stained cells was determined. 

To observe the migration of progenitor cells of adrenocytes, BrdU was injected before the beginning of the experiment. 14 days later, rats were sacrificed, and the samples were prepared and treated as described above. The migration of BrdU-positive cells was observed by microscopy at a magnification of 400x.

### 2.6. Measure of IGF-II mRNA Expression Using Quantitative PCR

Adrenocytes isolated from normal 4-month-old rats were cultured in RPMI 1640 and divided into a control group and an EF-treated group. In the latter, 10^−7^ M EF was added into RPMI 1640 and coincubated with adrenocytes for 24 hours. Then, cells were harvested and total RNA was isolated with Trizol reagent (Invitrogen, USA). Real-time two-step RT-PCR was performed with a QuantiTect SYBR Green PCR kit (Qiagen, Hilden, Germany) on an ABI GeneAmp 7300 Sequence detection system (Applied Biosystems, USA). The forward primer was 5′-TTGGCCAGATAAGGAGATGG-3′ the reverse primer was 5′-AGAGATGGCCCATAGGTGTG-3′. The parameters for PCR were the following: 50°C, 2 min; 95°C, 15 min; 35 cycles of 94°C, 10 sec; 55°C, 30 sec; 72°C, 30 sec, followed by hold at 72°C for 10 min. IGF-II mRNA was isolated from rat liver and reversed into cDNA as standard. Amplification specificity was confirmed by melting curve analysis using incorporated StepOne software v 2.0.

### 2.7. Statistical Analysis

Data were presented as the mean ± SD. Statistical analysis was by one-way ANOVA or unpaired *t-*test (two-tailed), and significance was assumed at *P* < 0.05.

## 3. Results

### 3.1. The Content of Epimedium Flavonoids (EFs)

**Figure 1:**
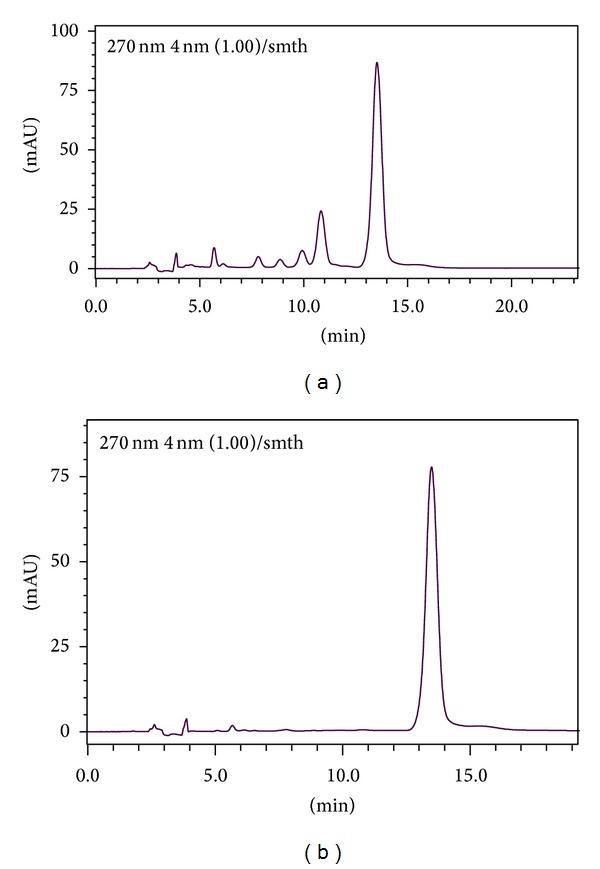
Content of epimedium flavonoids (EFs) by high-performance liquid chromatography (HPLC). (a) HPLC profiles of EFs; the content of two components is more than 10%. (b) HPLC profiles of standard samples of icariin. The content of icariin is 43.7% of the preparation.

The main ingredient of EFs was determined by high-performance liquid chromatography (HPLC). Results showed that there are two ingredients which contents are more than 10% (Figure 1(a)). Using the standard of icariin, we found the content of icariin accounts for up to 43.7% (Figure 1). Thus, our drugs are qualitatively controllable.

### 3.2. Effects of EFs on Body Weight Gain

**Figure 2:**
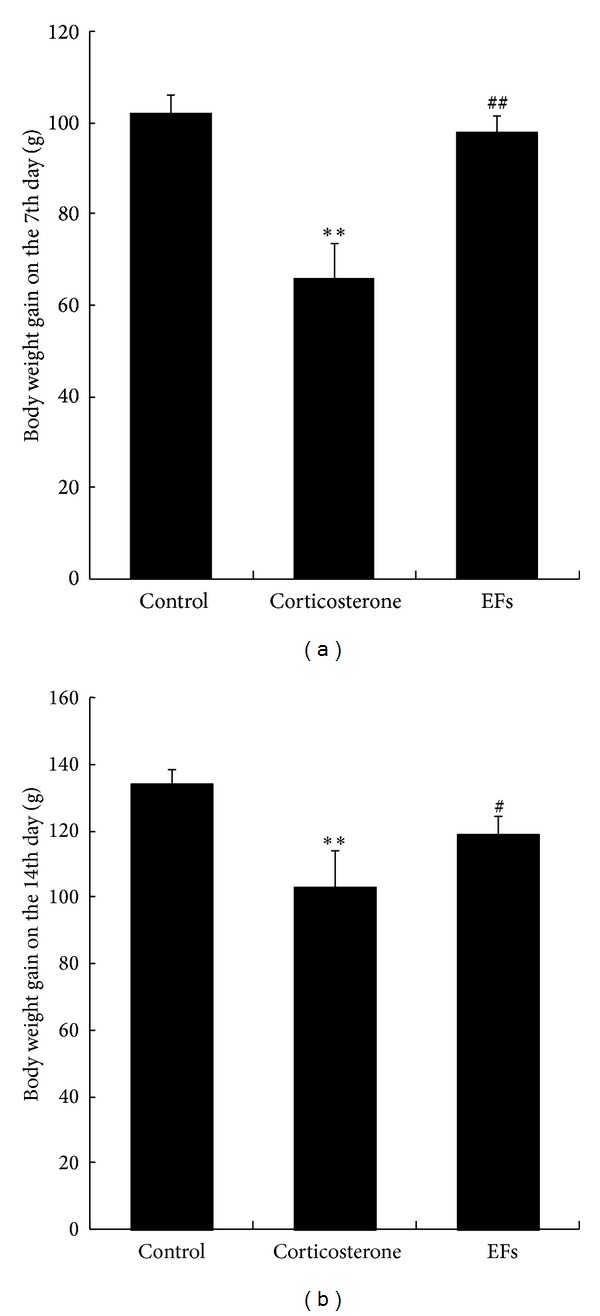
Effects of EFs on body weight gain of rats. (a) The body weight gain on 7th day for control, corticosterone only, and combined use of corticosterone and EFs was shown. (b) The body weight gain on 14th day for control, corticosterone alone, and combined use of corticosterone and EFs was shown. **P < 0.01 versus control group. ^#^ *P* < 0.05, ^##^ *P* < 0.01 versus corticosterone-only group.

The increase in body weight for control group on the 7th day was 102.25 ± 3.87 g. Corticosterone greatly delayed the growth of the rats (66 ± 7.52 g), suggesting a potent growth retardation effect of GCs. The weight gain in the combined corticosterone and EFs-treated rats was 98 ± 3.74 g on the 7th day, and the value was not statistically different when compared with control rats (*P* > 0.05) but significantly different (*P* < 0.01) when compared with corticosterone only-treated rats. These data suggest a protective effect for body weight of EFs in corticosterone-treated rats 7 days after treatment (Figure 2(a)).

On the 14th day, similar results were obtained (Figure 2(b)); body weight gains for control, corticosterone only-treated, and the combined corticosterone and EFs-treated group were 134.25 ± 4.54 g, 103.35 ± 10.85 g, and 119 ± 5.3 g, respectively. Although the gain in the combined corticosterone and EFs-treated group was lower than that in control rats, it was higher than that in corticosterone only-treated group (*P* < 0.05, versus control group and corticosterone only-treated group), indicating a partially protective effect of EFs on body weight gain after 14 days. 

### 3.3. Effects of EFs on Adrenal Gland Weight

Treatment with corticosterone significantly decreased organ weight of the adrenal gland; the value for control and corticosterone only-treated group was 49.63 ± 6.00 mg and 28.88 ± 9.45 mg, respectively. But in the combined corticosterone and EFs-treated group, the adrenal weight was increased to 34.97 ± 4.84 mg. When compared with corticosterone only-treated group, the difference was significant (*P* < 0.05).

### 3.4. Plasma ACTH and Corticosterone Level

**Figure 3:**
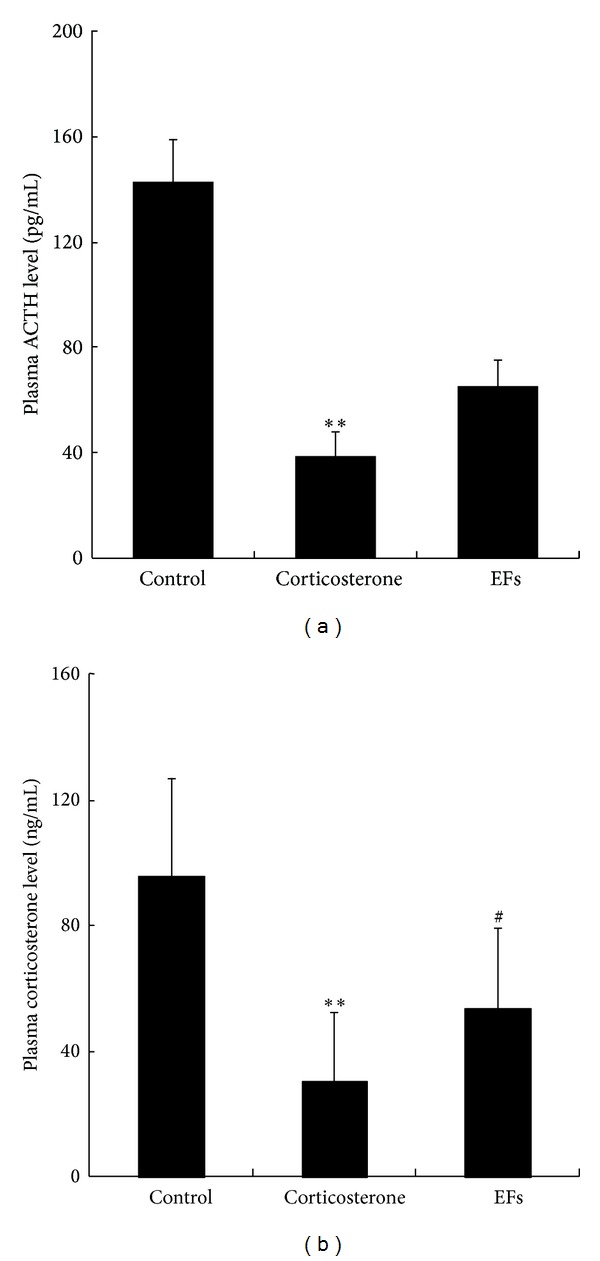
Effects of EFs on plasma ACTH and corticosterone level of rats. (a) Plasma ACTH level was measured by ELISA in control, corticosterone, and combined use of corticosterone and EFs. (b) Plasma corticosterone level was measured by ELISA after treatment with saline (control), corticosterone, and combined use of corticosterone and EFs. *P < 0.05, **P < 0.01 versus control group. ^#^ *P* < 0.05 versus corticosterone-only group.

The ACTH level in the control group was 143.15 ± 15.76 pg/mL, but in corticosterone only-treated group, the level was much lower at 38.9 ± 9.57 pg/mL. After treatment with combined corticosterone and EFs, there was a trend to an increase, but the difference was not significant when compared with the corticosterone only-treated group (Figure 3(a)).

The plasma corticosterone level in the control group was 96.03 ± 30.95 ng/mL. In corticosterone only-treated rats, the plasma corticosterone level was very low (30.72 ± 21.73 ng/mL), but after combined corticosterone and EFs treatment, the level (53.79 ± 25.63 ng/mL) of plasma corticosterone level was significantly elevated compared with the corticosterone only-treated group (*P* < 0.05), although the levels were still lower than that of control group (Figure 3(b)).

### 3.5. Progenitor Cell Proliferation in the Outer Layer of Adrenal Cortex

**Figure 4:**
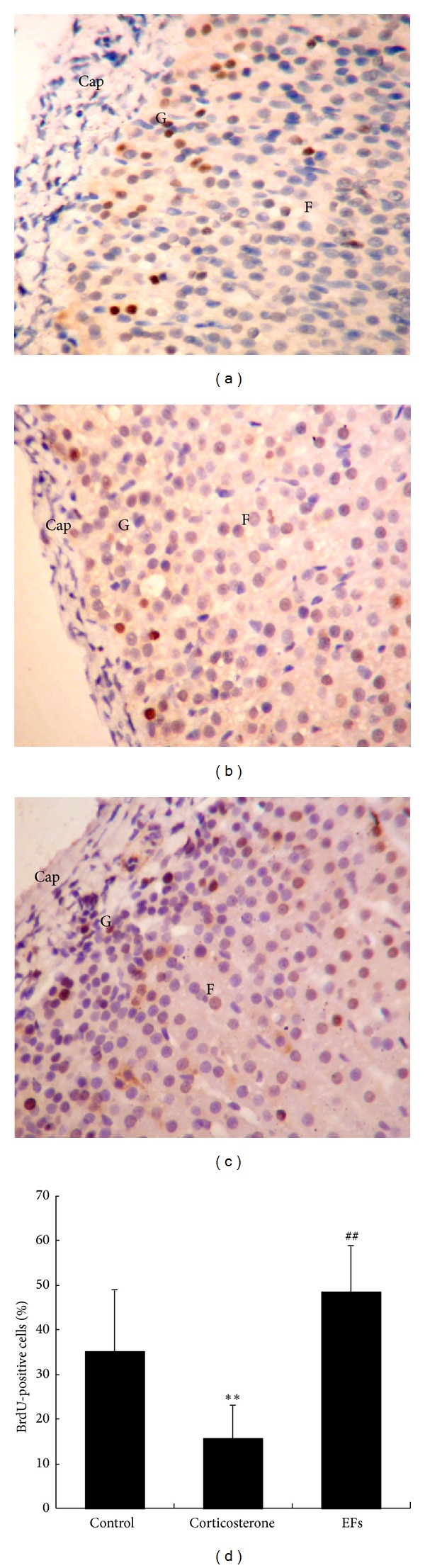
Localization and the number of BrdU-incorporated nuclei in the adrenal gland. ((a), (b), (c)) Sections of the adrenal gland from rats of the control group, corticosterone only-treated group, and combined corticosterone and EFs-treated group, respectively, were stained with anti-BrdU antibody and visualized with microscope (magnification ×400). (d) The percentage of BrdU-positive against total cells in zona glomerulosa was calculated. Cells with brown nuclei are considered as BrdU-positive cells. Cap, G, and F denote the capsule, zona glomerulosa, and zona fasciculata, respectively. All sections were counterstained with hematoxylin. **P < 0.01 versus control group. ^##^ *P* < 0.01 versus corticosterone-only group.

BrdU is an analog of uridine and is incorporated into nuclei of cells in which DNA synthesis is occurring; thus, by using a BrdU monoclonal antibody and immunohistochemical techniques, proliferating cells can be visualized. As shown in Figures 4(a), 4(b), and 4(c), BrdU-positive cells were concentrated in the outer layer of adrenal cortex in each group. After treatment with corticosterone, the percentage of BrdU-stained cells was decreased significantly (15.71 ± 7.58%). But in the combined corticosterone and EFs-treated rats, the percentage of BrdU-positive cells (48.52 ± 10.59%) was significantly increased (*P* < 0.01), when compared with the corticosterone only-treated group, and actually exceeded that in control group (35.15 ± 13.91%) (Figures 4(a), 4(b), 4(c), and 4(d)). These results suggest that EFs enhance proliferation of cells located in the outer layer of adrenal cortex.

### 3.6. Immigration of BrdU-Positive Cells toward the Inner Region of Adrenal Cortex

**Figure 5:**
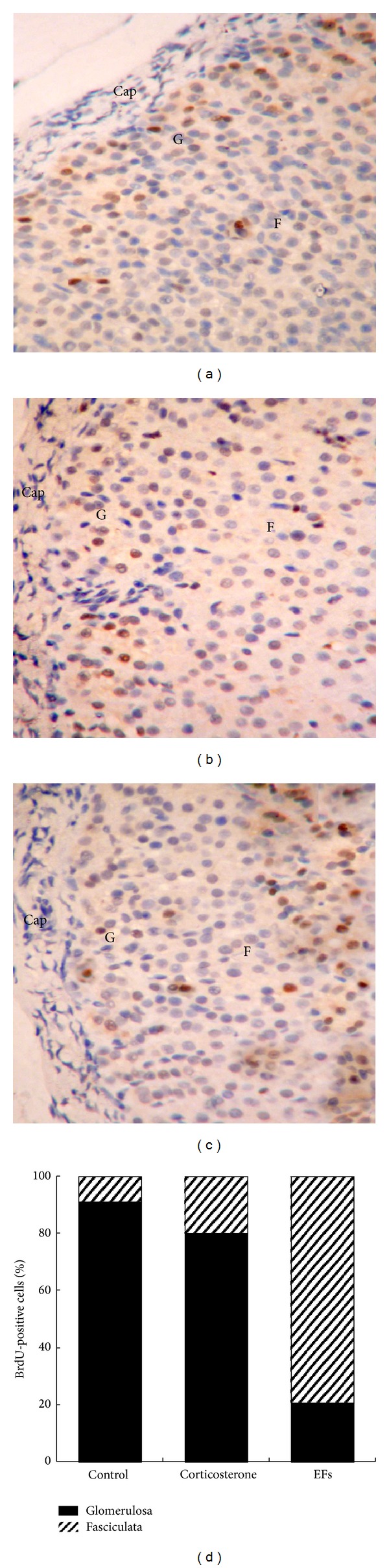
Migration of BrdU-positive cells toward the inner layer of adrenal gland. Rats were injected with BrdU; 14 days later, rats were killed and adrenal glands were excised, and the BrdU incorporation assay was carried out. ((a), (b), (c)) Sections of the adrenal gland from rats of the control group, corticosterone only-treated group, and combined corticosterone and EFs-treated group, respectively, were stained with anti-BrdU antibody and visualized with microscope (magnification ×400). (d) The percentage of BrdU-positive cells in zona glomerulosa or zona fasciculata against total BrdU-positive cells was calculated, respectively. Cells with brown nuclei are considered as BrdU-positive cells. Cap, G, and F denote the capsule, and zona glomerulosa, zona fasciculata, respectively. All sections were counterstained with hematoxylin.

To further investigate whether EFs affect the immigration of proliferating cells toward functional zones of adrenal cortex in the inner region to replenish the lost cells after corticosterone treatment, we injected rats with BrdU before the experiment, and then the rats were subjected to treatment with corticosterone only or combined corticosterone and EFs. After 14 days, immunohistochemical detection of BrdU was used to determine the migration of BrdU-stained cells. We found that most BrdU-positive cells in control and corticosterone only-treated rats still resided in the outer layer of adrenal cortex while the percentage of BrdU-positive cells residing in the fasciculata in control or corticosterone only-treated group was (8.8 ± 3.5)% and (20.0 ± 4.9)%, respectively. In the combined corticosterone and EFs-treated group, the majority of BrdU-positive cells (79.2 ± 11.6)% had migrated into the inner region corresponding to fasciculata zone of adrenal cortex, as shown in Figures 5(a), 5(b), 5(c), and 5(d), with *P* < 0.01, when compared with control or corticosterone only-treated group.

### 3.7. IGF-II mRNA Expression after Induction by EFs

Quantitative real-time analysis showed that EFs significantly upregulated the gene expression of IGF-II. In adrenocytes without addition of EFs, IGF-II mRNA was almost not detectable, with only (0.19 ± 0.24) × 10^3^ copies per 1 *μ*g initial total RNA. But after induction by EFs, the values were increased to (5.35 ± 3.52) × 10^3^ copies per 1 *μ*g initial total RNA, respectively, and thus increased more than 30-fold than that in normal adrenocytes. The experiment was repeated four times independently. Results exhibited statistical significance when EFs-treated adrenocytes were compared with adrenocytes without induction by EFs (*P* < 0.05).

## 4. Discussion

GCs suppress the HPA axis at various sites of the axis including the secretion of corticotropin (CRH) and ACTH. The suprahypothalamic brain regions such as the amygdala and the hippocampus are also involved in regulating the HPA axis [16–18]. In addition, long-term use of supraphysiological doses of GCs leads to atrophy of adrenal gland, due to a decrease of cell number, cell volume, and reduced cell proliferation in the adrenal cortex [19–21]. In the present study, we demonstrated that the plasma corticosterone levels decreased when rats were treated by corticosterone alone. But, after treatment with a combination of corticosterone and EFs, the levels of ACTH and plasma corticosterone were increased. Moreover, we found that EFs significantly counteracted the involution of the adrenal gland induced by corticosterone treatment. The results above suggest that EFs protect the HPA axis function from the effects of GCs. In the present study, the plasma ACTH level only showed a trend of an increase after combined corticosterone and EFs treatment without statistical significance. Dupouy et al. reported that heparin could slightly degrade the plasma ACTH level [22]. Whether the heparin interfered with the measurement of ACTH in our study remained unknown. But, this result might also suggest another possibility that there is an alternate mechanism(s) by which EFs exert their effects on maximally increased corticosterone levels, for instance, by acting on adrenocortex directly. 

To further investigate the cellular and molecular mechanisms by which EFs may counteract the atrophy induced by GCs, we performed cytogenesis and gene expression analysis in adrenal cortex. Results showed that EFs promoted the proliferation and migration inward of stem cells of adrenal cortex, suggesting a promoted regeneration of adrenal cortex by EFs. The adrenal cortex of mammals is composed of three morphologically and functionally distinct zones from the outer side to the inner side, the zona glomerulosa, the zona fasciculate, and the zona reticularis [23, 24]. The cell migration theory was proposed by Gottschau, where adrenocytes derive from the outer part of adrenal cortex, migrate centripetally, and differentiate into each functional zone [25]. Researchers confirmed that the precise compartment of the adrenocyte progenitors is located between the zona glomerulosa and zona fasciculata, and the newly divided cells migrate inwards [26]. This zone was identified as a stem cell zone for adrenal cortex [27], which builds a new field of adrenal cortex regeneration [28, 29]. The regeneration is characterised by cell proliferation, cell migration, and differentiation [30]. Therefore, we determined whether the cytogenesis of adrenal cortex can be enhanced by EFs. Consequently, BrdU-positive cells were confined to the subcapsular part of adrenal gland, consistent with previous reports [25]. We calculated the percentage of BrdU-positive cells to total cells to indicate the proliferation capability. Results showed that the percentage in the corticosterone-treated group decreased. But surprisingly, in rats simultaneously treated with GCs and EFs, there was an increased percentage of BrdU-positive cells, which exceeded that in normal rats, suggesting a strong proliferation-promoting property of EFs. We also analyzed the migration of BrdU-positive cells. In general, cells formed in the first compartment of zona glomerulosa traverse the second and into the innermost zona reticularis. Half of the cells are eliminated during the migration and the rest died in the reticularis by apoptosis [31]. A cell, which does not die during migration, will take about 104 days to reach the reticularis [25]. Here, we found that most BrdU-positive cells either in control rats or GCs only-treated rats remained in the zona glomerulosa, perhaps due to too short time interval than 104 days to observe the migration. In contrast, in the rats treated with GCs and EFs, most of BrdU-staining cells migrated into zona fasciculata, suggesting increased migration.

Numerous studies established that ACTH is the major regulator of the adrenal cortex. Upon the use of GCs, ACTH can counteract the atrophy of adrenal gland through enhanced proliferation and migration of adrenocytes between zona glomerulosa and zona fasciculata [32, 33]. Recent studies have provided a new concept that the adrenocortex is also regulated by autocrine and/or paracrine factors including IGFs [34, 35], fibroblast growth factors (FGFs) [36], endothelin [37], cholecystokinin [38], adrenomedullin [39], and cytokines [40]. Among these, IGF II is the most important and is involved in cell division, steroid synthesis, and cell differentiation of adrenocytes [41]. Thus, we measured the IGF II gene expression using isolated adrenocytes coincubated with EFs for a specified time. Results showed that EFs significantly upregulated IGF II gene expression. In our study, the increase of ACTH by EFs treatment was not statistically significant. This unexpected result may be due to the interference of heparin. Thus, our data do not preclude a role for ACTH in mediating effects of EFs. We believe that our data are consistent with EFs exerting their effect on adrenocytes through both ACTH and the autocrine and/or paracrine factor IGF II.

Another striking result was that EFs markedly counteracted the decrease of body weight gain induced by corticosterone, which is the consequence of the nonspecific catabolic effect of the steroid. There are reports which provide evidence that thyroid hormone or growth hormone administration could prevent the decrease in body and organ weight gains [42]. In the present study, we showed that EFs upregulated IGF II, which is a component of growth hormone-/insulin-like growth factor (GH/IGF) axis. Increased body weight gain as a result of EFs treatment may be due to elevated GH/IGF axis activity. But the precise molecular mechanism needs to be further investigated.

According to our experimental design, the EFs treatment on normal rats was omitted. Thus, there was a possibility that effects of EFs were achieved through altering the pharmacokinetics of the oil or corticosterone. However, considering our accumulated data, we believe that these effects were induced by EFs. For example, IGF II mRNA was significantly increased by more than 30 fold, which can be attributed to specific pharmacological effects of EFs. In summary, EFs extract from a herbal origin proved to be effective against the suppressive effects of GCs. EFs could be considered for clinical use to selectively counteract the side effects of GCs on the HPA axis, and thus provide an effective and safe alternative treatment.
